# Combined systematic screening for malnutrition and dysphagia in hospitalized older adults: a scoping review

**DOI:** 10.1186/s12877-024-05070-6

**Published:** 2024-05-21

**Authors:** Susanne M. Javorszky, Christoph Palli, Susanne Domkar, Bernhard Iglseder

**Affiliations:** 1 Institute of Nursing Science and Research, Paracelsus Medical University, Strubergasse 21, 5020 Salzburg, Austria; 2 FH Joanneum, Institute of Health and Nursing, Alte Post Straße 149, 8020 Graz, Austria; 3 FH Campus Wien, Department of Health Sciences, Favoritenstraße, 226, 1100 Vienna, Austria; 4 Department of Geriatric Medicine, Christian-Doppler-Klinik, Paracelsus Medical University, Ignaz-Harrer-Straße 79, 5020 Salzburg, Austria

**Keywords:** Dysphagia, Presbyphagia, Malnutrition, Screening, Assessment, Elderly, Geriatric

## Abstract

**Background:**

Dysphagia affects about 40% of patients admitted to acute geriatric wards, as it is closely associated with diseases that rise in prevalence with advancing age, such as stroke, Parkinson’s disease, and dementia. Malnutrition is a highly associated predictive factor of dysphagia as well as one of the most common symptoms caused by dysphagia. Thus, the two conditions may exist simultaneously but also influence each other negatively and quickly cause functional decline especially in older adults. The purpose of this review was to determine whether institutions have established a protocol combining screenings for dysphagia and malnutrition on a global scale. If combined screening protocols have been implemented, the respective derived measures will be reported.

**Methods:**

A scoping review was conducted. A systematic database search was carried out in January and February 2024. Studies were included that examined adult hospitalized patients who were systematically screened for dysphagia and malnutrition. The results were managed through the review software tool Covidence. The screening of titles and abstracts was handled independently by two reviewers; conflicts were discussed and resolved by consensus between three authors. This procedure was retained for full-text analysis and extraction. The extraction template was piloted and revised following feedback prior to extraction, which was carried out in February 2024.

**Results:**

A total of 2014 studies were found, 1075 of which were included for abstract screening, 80 for full text screening. In the end, 27 studies were extracted and reported following the reporting guideline PRISMA with the extension for Scoping Reviews.

**Conclusion:**

Most of the studies considered the prevalence and association of dysphagia and malnutrition with varying outcomes such as nutritional status, pneumonia, oral nutrition, and swallowing function. Only two studies had implemented multi-professional nutrition teams.

## Introduction

The World Health Organization (WHO) defines “older people” as 60–75, “old people” as 76–90, “very old people” as 91–100 and “oldest-old” or “centenarians” as people aged over 100 years [1]. Still, the definition of “older people” varies based on cultural, social, functional, and emotional factors. However, demographic changes contribute to an increasing proportion of individuals around and after retirement age. In the European Union, individuals aged 65 and over are considered “older people,” with a projected 28.5% share of the total EU population in 2050 [2]. This demographic shift poses significant challenges to the healthcare systems, given the association between aging and various health conditions such as diabetes, cardiovascular disease, Parkinson’s disease, stroke, and dementia. This challenge is compounded by the simultaneous retirement of baby boomers and a pronounced shortage of skilled workers in nursing and therapeutic care. Consequently, healthcare systems face the urgent need to formulate efficient, resource-saving, and high-quality care strategies for older adults in general and specifically for geriatric patients.

Aside from chronological aging, differences in functioning and subjective health lead to a need for a functional and multidimensional definition of “geriatric patients” [3, 4]. Physiological aging affects cellular processes, which generally impacts nutrient metabolism as well as muscle function, thus changing the swallowing function physiologically, leading to an aged swallow called presbyphagia [5, 6]. There is a high correlation between frailty, dysphagia and malnutrition, highlighting the importance of these conditions in the context of advancing age, accumulated burden of disease and frailty [7, 8]. Advancing age raises the risk of malnutrition, either as a direct result of diseases as well as multimorbidity, or caused by challenges related to food intake itself, particularly issues with chewing and swallowing [9, 10]. Insufficient supply of nutrients leads to a state of malnutrition, which in turn has a negative effect on all other health factors. It has been shown that frail older individuals benefit from individualized nutritional support. The achievement of energy and protein goals is essential for improved outcomes [11]. The use of fortified meals and oral nutritional supplements with > 400 kcal and at least 30 g of protein / day is recommended if the intake through meals is not sufficient [12]. Dysphagia (swallowing disorder), which impairs the ability of processing and swallowing food and liquids, is one of the most significant predictors of malnutrition and is in turn negatively influenced by it [13–15].

## Background

In 2016, the European Society of Swallowing Disorders (ESSD) declared dysphagia a geriatric syndrome that is multifactorial and has significant negative impact on the nutritional and health status of geriatric patients in general [16]. The prevalence of dysphagia in older adults ranges considerably, with reported rates between 10 and 90% in nursing homes and a pooled prevalence of 35,9% as reported in a recent meta-analysis by Tian et al. [17], while Doan et al. [18] report a pooled prevalence of 47%. As dysphagia is closely associated with frailty as well as neurodegenerative diseases, the prevalence will increase in institutions specialized in caring for older persons with advanced stages of diseases as well as higher age and higher rates of frailty [7]. The estimated prevalence in hospitalized older adults is 37% and in community dwelling older adults around 18% [18]. In addition to a loss of quality of life due to possible restrictions in food intake [19–21], one of the most serious consequences of dysphagia is malnutrition [8]. In individuals with a compromised immune system and poor oral health dysphagia can lead to the development ofaspiration pneumonia [22, 23], which has a significantly higher mortality rate than pneumonia of other etiologies [24, 25]. The diagnosis of dysphagia is carried out in several steps, usually starting with an initial screening to identify high-risk patients. This first screening can be completed by different professions [26]. If a patient is determined to be at risk, a referral is made to a clinical swallow examination carried out by a speech language therapist (SLT). This clinical assessment may result in the recommendation of an instrumental diagnosis of the swallowing disorder using FEES (flexible endoscopic evaluation of swallowing) or VFSS (videofluoroscopic swallowing study), which provides foundation for individualized treatment planning. Dysphagia treatment is carried out by means of speech language therapy. It may take place in a rehabilitative manner through targeted exercises, in a compensatory manner by developing strategies to optimize the swallowing function, and adaptively through the use of dietary modification and adaptation of tableware. These adaptations can include the recommendation ofspecific cups and cutlery as well as optimal positioning of patients when eating or drinking. Dysphagia management should also include dietetic and nutrition therapy [16, 27, 28].

Particularly for older people, malnutrition is a serious health problem with far-reaching effects on their quality of life and health [9, 29]. Inadequate nutrient intake is associated with increased susceptibility to infection, extended recovery time, reduced muscle mass, a weakened immune system, and a higher risk of falls [30]. The diagnosis of malnutrition usually follows similar steps to those for diagnosing dysphagia, starting with rapidscreenings. A variety of screening and assessment methods are used in conjunction with blood parameters as well as measurements of body parameters such as weight, circumference of limbs or measuring skinfold thickness [31]. The treatment of malnutrition in view of determining needs and optimizing nutrient intake is carried out by dietitians.

Severe cases of both dysphagia and malnutrition can be treated with promising results using a combined treatment approach [32]. Numerous screening methods are endorsed internationally for early detection for both conditions. These methods are widely integrated into the healthcare of older individuals and explicitly recommended for use [33–35].

Due to the close association of the two disorders, assessing them in a combined screening procedure seems a promising strategy. Therefore, it might be feasible to detect both with one quick screening protocol. A positive screening result should lead to timely referrals to either speech language therapists (SLT) for dysphagia assessment and treatment or to registered dietitians (RD) for malnutrition assessmentand treatment. This consideration led to the decision to conduct a literature review with the aim of ascertaining whether internationally combined screenings for malnutrition and dysphagia already existed. In this case, follow-up questions were defined. Which specific screening tools were used by which profession? Which specific outcomes of an implemented combination screening were measured? The research question was structured according to the PICO scheme (Table 1). Since the question implies a heterogeneity of studies to be processed and possible outcome measures of the individual studies, the methodological approach of a scoping review according to the recommendations of the PRISMA-Scr statement was chosen. The large number of possible outcomes defined in the PICO question led to the decision to conduct a scoping review, which is particularly suitable for obtaining an initial overview of a topic and for broadly reflecting the state of research without narrowing the included results too much [36, 37]. For guidance on the methodology, several sources were used [37–39].

**Table 1:** PICO Question

PICO: What are the outcomes of an implemented combined screening of swallowing function and nutritional status in hospitalized patients aged 60 years and older?	
Population	Patients ≥ 60yrs at hospitalization
Intervention	combined screening of swallow function and nutritional status conducted by specific profession
Comparison	No systematic screening
Outcomes	Pneumonia, fall, rehospitalization, length of stay, place of discharge, nutritional status, wound or bone healing, mobility, oral nutrition, diet consistency, oral medication, bolus death

## Methods

An informal protocol was developed which included the defined search terms, research question, time schedule, task distribution amongst the authors, selected databases, selection criteria, and the search strand based on MeSH terms. This protocol was not published and merely facilitated orientation in the authors’ work process. Furthermore, the review protocol has not been registered in any official database.

The terms for the search strand were: presbyphagia, dysphagia, swallowing disorder, *nutrition*, sarcopenia*, weight loss, diet*, assessment, screen*, elderly, hospital admission, geriatric, nursing home, nursing facility, long term care. The asterisks stand as so-called “wildcard” symbols, which allow any stem ending of the word to be included. Using the completed search strands, the databases Science Direct, CINAHL, PubMed, Google Scholar, MedLine and Cochrane Library (Trials) were searched between 31.1.2024 and 21.2.2024. Previously published quantitative primary studies were included, which systematically recorded both parameters (dysphagia and malnutrition) in hospitalized adults. The exclusion criteria were an average age of less than 60 years, patients undergoing head and neck cancer surgery as well as persons in other healthcare settings. Furthermore, studies in which only one of the two parameters had been systematically recorded were excluded, as well as studies in which instrumental diagnostics were carried out instead of a screen. The decision to focus only on intramural care pathways was made due to the increasing heterogeneity of the professional groups and healthcare systems involved. In addition, studies that not published in German or English were excluded. There was no restriction on publication date.

For literature management, the web-based collaborative software platform tool Covidence was used to support the creation of literature reviews developed by Veritas Health Innovation [40]. Duplicates were automatically removed. Two authors rated the remaining studies for inclusion after screening all titles and abstracts with SMJ screening and CP and SD acting as second raters. Conflicting ratings were solved by consensus of SMJ, CP and SMD. In most cases, the main reason for different ratings was the reason for exclusion, as some studies met several of the exclusion criteria at the same time.

In the next step, a full-text review was carried out using Covidence following the same procedure as the title and abstract screening. Again, two authors had to give an independent evaluation; conflicts were solved by consensus between SMJ, CP and SD.

For data extraction, an extraction template was created in Covidence by SMJ, which was based on the PICO question. The template was piloted by CP and SD each in one study. Following feedback, it was revised and used for the extraction of all remaining studies as of February 2024 (Table 2). Extraction was carried out independently by two authors, either SMJ and CP or SMJ and SD. After extracting 13 studies, a meeting was held to avoid disparities in abbreviations (e.g. different use of commas and full stops leads to the software declaring a conflict) and formal details as differences were noticed when reporting studies details such as time frames by having been written as “January, 2015 – June, 2016” compared to “01/2015–06/2016” for example In another meeting following the extraction, all conflicts and open questions were discussed and solved based on consensus among the authors.

**Table 2:** Data extraction template

**Data Extraction Template**	
**Variable**	**Possible Answers**
Study ID	Short Receipt
Title	
First author and correspondence data	
Country of study	United States; UK; Canada; Australia/NZ; Spain; Brazil; Portugal; Sweden; Norway; Japan; Korea; Other
Aim of the study	
Study design	RCT; Non-randomized experimental trial; cohort study; Cross-sectional Stude; Case Control Study; prevalence study; case series; case report; diagnostic study; Economic Evaluation; Other
Start of study	
End of study	
Population studied	
Age of the subjects	
Setting	
Inclusion Criteria	
Exclusion criteria	
Total number of subjects	
Dysphagia screening tool used	
Screening tool used for malnutrition	
Professional Group Dysphagia Screening	
Executing professional group Malnutrition screening	
Are the two screenings implemented permanently?	Yes; No; Other
If so, how long have the screening measures been in place?	
Measures derived from screening results	Referral to speech therapy; referral to registered dietitian; deciding on oral nutrition vs. NPO; deciding on diet/consistency; Other
Repetition of the screening during the stay	Yes, depending on the period; Yes, after intervention; Yes, after discharge; No; Other
Nutrition team in place	Yes; No
If there is a nutrition team—professional groups involved	
Nutritional counseling is part of discharge management	Yes; No; Unclear; Not suitable; Other
Outcomes	Pneumonia; Fall; Rehospitalization; Length of stay; Place of discharge; Nutritional status; Wound and bone healing; Mobility; Sufficient oral intake; Oral medication; Bolus death; Other

The heterogeneity of the study types processed and the methodological approach within the framework of a scoping review resulted in avoiding a critical assessment of the level of evidence and the bias risks of the individual studies [37–39]. The extracted data was exported to Microsoft Excel for an easier overview, comparison of the extracted information and to generate the tables.

## Results

**Fig. 1:**
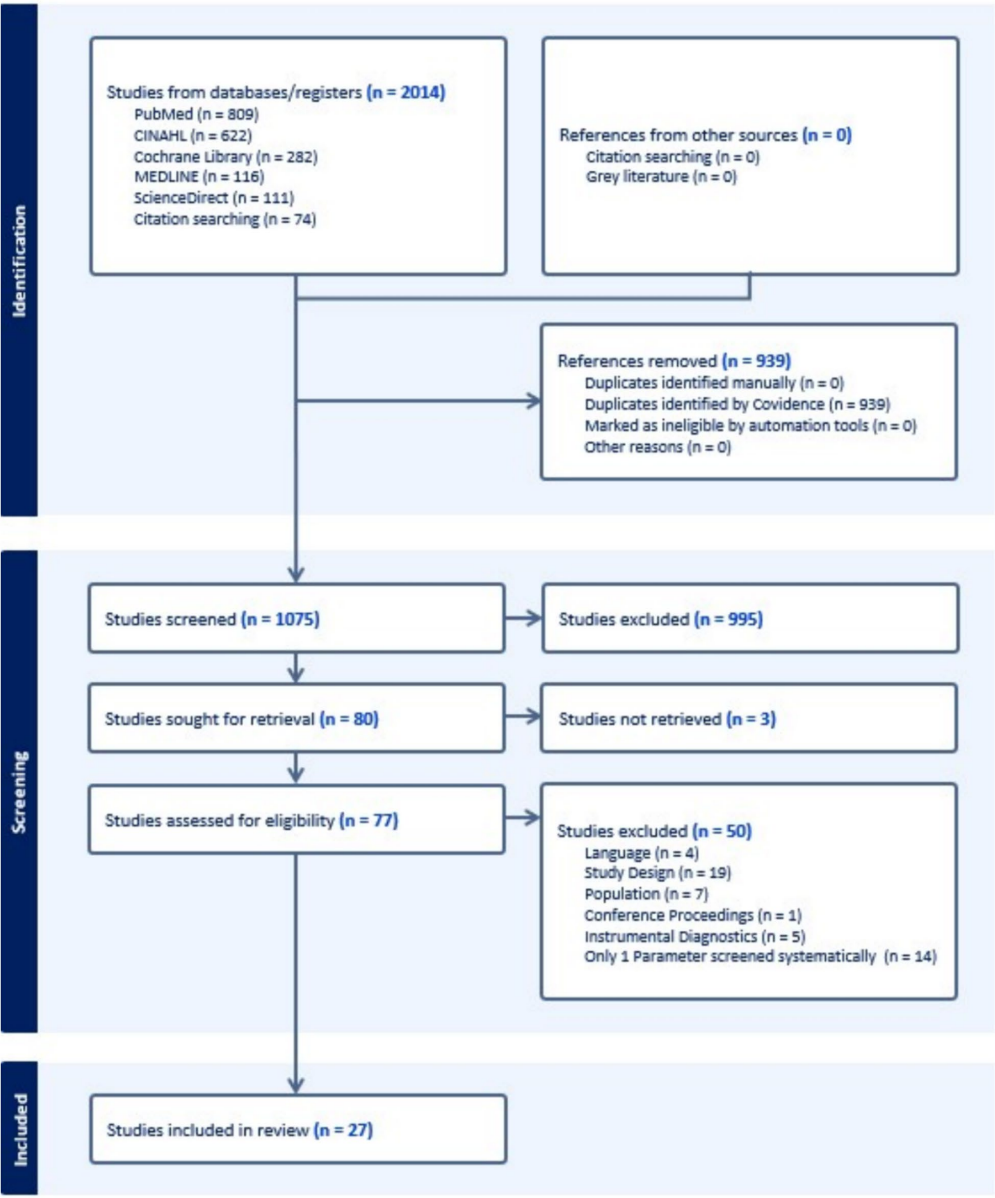
PRISMA flowchart

As shown in Fig. 1, a total of 2014 studies were retrieved from database search, 939 of which were duplicates that were removed. The remaining 1075 studies were independently evaluated by two authors based on title and abstract. 995 studies were excluded. 77 studies were assessed for inclusion based on their full text, which in turn was independently conducted by two authors. A total of 50 studies were excluded for the following reasons: language (4), study design (19), population (7), conference proceedings (1), instrumental diagnostics (5) or only 1 parameter screened systematically (14).

Data was extracted from theremaining 27 articles using the piloted template. Table 3 gives an overview of the characteristics of all included studies. Only three of the included studies were older than 10 years, 12 studies were conducted between 2014 and 2019 and 12 studies were conducted since 2020 with the most recent dated 2023. The settings of the studies vary, but the vast majority was conducted in acute care hospitals (18), specifically in acute geriatrics (3). Three studies investigated patients in rehabilitation centers (3). Three studies were multicentric and examined populations from different settings (3). The total number of participants varied considerably, from small cohorts of 12 to large-scale investigations with over 1,000 participants.

**Table 3:** Study characteristics

Study ID	Country	Goal	Age	Setting	n
Alvarez-Larruy et al. 2023 [41]	ESP	Comparison between SFF, salivary Substance P, hydration and nutritional status	72 +—13.5	AH	45
Andrade et al. 2018 [42]	BRA	Incidence of Dysphagia and Associated Factors, Nutritional Status—in Correlation with EAT-10	54 SD 20.2	AH	909
Cabre et al., 2010 [43]	ESP	Prevalence of Clinical Signs of Aspiration and their Predictive Value in Terms of 30-day and 1-year Mortality	≥ 70	AG	134
Carrión et al. 2015 [44]	ESP	Association Between Oropharyngeal Dysphagia, Nutritional Status and Clinical Outcome in Elderly Patients Admitted to Acute Geriatrics	≥ 70	AG	1662
Mateos-Nozal et al. 2020 [45]	ESP	Prevalence and Main Risk Factors for Dysphagia	93.5 SD 4.1	AH	329
Crary et al. 2006 [10, 46]	USA	Prevalence of Dysphagia and Poor Nutritional Status in Patients with Acute Ischemic Insult, as well as Identify Potential Associations with the Severity of the Insult and these two Outcomes	66.2 SD 11.8	AH	76
Donini et al. 2011 [47]	ITA	Prevalence and Associated Factors of Anorexia in the Elderly; Assessment of the Influence of Senile Anorexia on Nutritional Status, Eating Habits and Functional Status	≥ 65	LTC, RF, AG, CD	526
Drozdz et al. 2014 [48]	BRA	Effect of Speech Therapy on the Severity of Dysphagia, Nutritional Status, Anxiety and Oral Nutrition	64.6 M	AH	12
Manas-Martinez et al. 2018 [49]	ESP	Prevalence of Oropharyngeal Dysphagia by EAT-10 and its Association with Malnutrition and Long-Term Mortality	83 SD 11.8	AH	90
Matsuo et al. 2017 [50]	JPN	Prevalence of Dysphagia, Association of Dysphagia and Functional Status	80.5 SD 7.9	AH	103
Moncayo-Hernandéz et al. 2023 [10]	COL	Prevalence of Sarcopenic Dysphagia	≥ 65	LTC	100
Namasivayam-MacDonald et al. 2017 [42]	CAN	Association between Nutritional Status, Eating Habits, Clinical Signs of Dysphagia and Tongue Strength	86.8 SD 7.83	LTC	639
Olesen et al. 2021 [13]	Code	Prevalence of Dysphagia in Geriatric Patients	≥ 65	AH	334
Omura et al. 2021 [51]	JPN	Association between Swallowing Screen and Aspiration Pneumonia	83.8 SD 8.5	LTC	39
Ortega Barrio et al. 2019 [52]	ESP	Association between Malnutrition and Dysphagia in Insult	34–94	AH	183
Poisson et al. 2016 [53]	FRA	Associations between Oral Health, Dysphagia and Malnutrition in Hospitalized, Elderly Patients	85.28 SD 5.68	AH	159
Popman et al. 2018 [14]	NZL	Prevalence of Dietary Risk and Associated Risk Factors	90 +—3.7	AH	88
Ramos-Vázquez et al. 2021 [54]	MEX	Association Between Cachexia, Muscle Strength and Dietary Risk with Dysphagia	73 M	AH	79
Shimazu et al. 2020 [10]	JPN	Frequency of Referrals for Individualized Nutritional Therapy in Post-Stroke Patients Undergoing Rehabilitation, as well as the Impact on Outcomes in ADLs, Length of Hospital Stay, and Dysphagia	71.8 SD 13.3	RF	426
Tanigör et al. 2020 [55]	TUR	Prevalence of Nutritional Deficiencies and Dysphagia in Patients with and without Sarcopenia	57.2 M	RF	128
Tran et al. 2021 [14]	VNM	Prevalence and Association between Malnutrition and Dysphagia	75.5 SD 7.2	AH	1007
Uno et al. 2020 [56]	JPN	Effect of Improved Nutritional Status on ADLs and Dysphagia	84.7 SD 7.8	AH	143
Vidal Casariego et al. 2020 [57]	ESP	Usability of the EAT-10	76 SD 17.9	AH	196
Wakabayashi 2016 [58]	JPN	Association between Dysphagia, Nutritional Status and ADL's	82 M	AH, LTC	237
Waza et al. 2019 [59]	JPN	Influence of the Kuchi-kara Taberu index on the Course of Rehabilitation	76.4 SD 12.3	RF	233
Wham et al. 2017 [60]	NZL	Prevalence of Malnutrition in the Elderly	80.4 SD 8.7	AH, LTC, CD	167
Yu et al. 2024 [61]	CHN	Relationship between self-perceived oral health, self-reported nutritional status, dysphagia and frailty	68–78	AH	980

*Legend*: *AH* Acute Care Hospital, *AG* Acute Geriatric Ward, *LTC* Long-Term Care, *RF* Rehabilitation Facility, *CD* Community Dwelling; Countries by International Abbreviations (ISO); Age as specified, *M* mean, *SD* Standard Deviation, *SFF* Spontaneous Swallowing Frequency

All studies investigated the association between nutritional status and dysphagia in older people in cohort and cross-sectional studies. The studies include participants from Japan (6), Spain (7), New Zealand (2), Brazil (2), Denmark (1), France (1), Italy (1), Canada (1), Colombia (1), Mexico (1), Turkey (1), the United States (1), China (1) and Vietnam (1). The parameters studied vary from the prevalence of malnutrition and nutritional risks to the influence of speech therapy on dysphagia and associations between oral health, dysphagia, and malnutrition. The age groups range from over 65 years to over 70 or 75 years and older. The main characteristics of the described studies are shown in Table 3.

### Dysphagia screening

As per the inclusion and exclusion criteria, any bedside tool to detect swallowing difficulties was included under the broad term “dysphagia screening”. The term “screening” is generally understood to mean an easy to apply early detection protocol to identify individuals of risk of a specific disorder. A positive screening result, suggesting a possible risk for, in this case, a swallowing disorder, warrants further, more detailed assessment by specialized professionals [62]. Since dysphagia diagnostics can only be completed with an instrumental evaluation, any bedside assessment can be classified as a form of screening with differing sensitivity and specificity depending on the variables included and the amount of training needed to conduct the screening. Rommel & Hamdy describe the dysphagia detection process as a multi-step approach beginning with any screening. In case of a positive screening result, a clinical swallow assessment needs to be conducted by a dysphagia specialist, usually a speech language therapist (SLT). This assessment includes the patient history, a physical and oral motor exam as well as a food intake assessment [63]. This description illustrates the argument, that also protocols referred to as “assessments” like the Volume-Viscosity Swallowing Tool (V-VST) [64] can be defined as screening tools in the process of dysphagia detection. For this scoping review the authors decided to include the Swallowing-related Quality of Life Tool (Swal-QoL) [65] as well, as self-reported limitations in the quality of life because of swallowing disorders can be counted as proxy parameter for existing dysphagia.

The most frequently used screening tool for dysphagia was the self-reported Eating Assessment Tool (EAT-10) [66], which was used in eight studies as the sole tool and in three more in a combination of two or more tools. The second most commonly reported screening tool was the Volume-Viscosity Swallowing Test (V-VST) [64]. Table 4 gives an overview of all the 11 reported screening tools used to assess dysphagia in the 27 studies.

**Table 4:** Tools reported in the studies to screen for dysphagia

Study ID	Screen Dysphagia
Alvarez-Larruy et al. 2023 [41]	V-VST
Cabre et al., 2010 [43]	WST
Carrión et al. 2015 [44]	V-VST
Mateos-Nozal et al. 2020 [45]	V-VST
Andrade et al. 2018 [42]	EAT-10
Crary et al. 2006 [10, 46]	MASA, FOIS
Donini et al. 2011 [13]	SWAL-QOL
Drozdz et al. 2014 [48]	PARD, FOIS
Manas-Martinez et al. 2018 [49]	EAT-10
Matsuo et al. 2017 [50]	EAT-10
Moncayo-Hernandéz et al. 2023 [10]	V-VST
Namasivayam-MacDonald et al. 2017 [42]	STAND
Olesen et al. 2021 [13]	EAT-10, WST (GUSS)
Omura et al. 2021 [51]	MASA
Ortega Barrio et al. 2019 [52]	V-VST
Poisson et al. 2016 [53]	WST
Popman et al. 2018 [14]	EAT-10
Ramos-Vázquez et al. 2021 [54]	EAT-10, V-VST
Shimazu et al. 2020 [10]	FILS
Tanigör et al. 2020 [55]	EAT-10, FOIS, MDADI
Tran et al. 2021 [14]	EAT-10
Uno et al. 2020 [56]	FILS
Vidal Casariego et al. 2020 [57]	EAT-10
Wakabayashi & Matsushima 2016 [58]	EAT-10
Waza et al., 2019 [59]	FOIS
Wham et al. 2017 [60]	EAT-10
Yu et al. 2024 [61]	EAT-10, WST

Legend: *EAT-10* Eating Assessment Tool, *FOIS* Functional Oral Intake Scale, *FILS* Food Intake Level Scale, *GUSS* Gugging Swallowing Screen, *MASA* Mann Assessment of Swallowing Ability, *MDADI* M.D. Anderson Dysphagia Inventory, *PARD* Protocol of Risk Assessment for Dysphagia, *STAND* Screening Tool for Acute Neurological Dysphagia, *SWAL-QOL* Swallowing Related Quality of Life, *WST* Water Swallowing Test, *V-VST* Volume-Viscosity Swallowing Test

The professional groups carrying out the screenings were reported in only 6 studies (Nursing staff/study nurse: Cabre et al. 2010; Carrión et al. 2015; Mateos-Nozal et al. 2017; Shimazu et al. 2020; SLT: Crary et al. 2006; Moncayo-Hernandéz et al. 2023) [10, 43–46, 49]; 7 studies used exclusively self-reported tools (Manas-Martinez et al. 2018; Matsuo et al. 2017; Popman et al. 2016; Tran et al. 2021; Vidal Casariego et al. 2020; Wakabayashi & Matsuhima 2016; Wham et al. 2017) [41, 47, 50, 57, 58, 60, 67] and the remaining 14 did not specify the profession [13, 42, 48, 51–56, 59, 61, 68–70].

### Malnutrition screening

Malnutrition screening was carried out by Mini Nutritional Assessment (MNA) [71] in the majority of studies having been reported 20 times, two times in the long form, 11 times in the short form and seven times without further specification. Two studies used the Geriatric Nutritional Risk Index (GNRI) [72] and the remaining two used the Subjective Global Assessment (SGA) [73].

Even in the case of malnutrition screening, information on the occupational group performing the tests was found in a minority of studies. Two studies reported registered dietitians as responsible [44, 67], one named speech language therapists [10], one a multi-professional team [43], and two described nursing staff as being responsible with one of them explicitly mentioning a study nurse [45, 46].

#### Interventions taken after screening

Only two of the reviewed studies provided clear information on the long-term implementation of the two screenings with Waza et al. using the FOIS and the GNRI [69] and Uno et al. using the FILS and the MNA-SF [68]. Both describe the screening process as part of routine patient care and report on specific measures derived from the screening results, such as a referral to swallowing of nutritional specialists or a recommendation on texture for oral nutrition. Five studies reported that screening was systematically repeated, either after an intervention, after a certain amount of time or right before discharge [51, 55, 56, 68, 69]. Table 5 provides an overview of the subsequent intervention following positive screening results.

**Table 5:** Overview of reported interventions following positive screening results

Study ID	Measures
Cabre et al., 2010 [43]	N/A
Carrión et al. 2015 [44]	N/A
Mateos-Nozal et al. 2020 [45]	Oral Nutrition vs. NPO Decision; Decision TMD; Advice on Positioning
Andrade et al. 2018 [42]	N/A
Crary et al. 2006 [10, 46]	N/A
Donini et al. 2011 [47]	N/A
Drozdz et al. 2014 [48]	Referral to SLT; Oral Nutrition vs. NPO Decision; Decision on TMD
Manas-Martinez et al. 2018 [49]	N/A
Matsuo et al. 2017 [50]	N/A
Moncayo-Hernandéz et al. 2023 [10]	Referral to SLT
Namasivayam-MacDonald et al. 2017 [42]	N/A
Olesen et al. 2021 [13]	N/A
Omura et al. 2021 [51]	N/A
Ortega Barrio et al. 2019 [52]	N/A
Poisson et al. 2016 [53]	Decision on TMD
Popman et al. 2018 [14]	N/A
Ramos-Vázquez et al. 2021 [54]	Oral Nutrition vs. NPO Decision; Decision on TMD;
Shimazu et al. 2020 [10]	Referral to SLT; Referral to RD
Tanigör et al. 2020 [55]	N/A
Tran et al. 2021 [14]	Decision on TMD
Uno et al. 2020 [56]	Referral to SLT, Referral to RD, Nutritional Therapy
Vidal Casariego et al. 2020 [57]	N/A
Wakabayashi et al. 2016 [58]	N/A
Waza et al., 2019 [59]	Referral to SLT, Referral to RD, Oral Nutrition vs. NPO Decision, Decision on TMD, Oral Health Care Plan, Respiratory Therapy, Review of Medication
Wham et al. 2017 [60]	N/A
Alvarez-Larruy et al. 2023 [41]	Decision on TMD
Yu et al. 2024 [61]	N/A

*Legend*: *NPO* Nil Per Os, *SLT* Speech Language Therapy, *TMD* Texture Modified Diet, *RD* Registered Dietitian

While only three studies explicitly indicated nutritional therapy or referral to registered dietitians as an intervention, nutritional status is the most frequently reported outcome of the studies and was defined as such in all studies except Moncayo-Hernandéz et al. [49] and Omura et al. [54], i.e., a total of 25. The other two most common outcomes were sufficient oral intake and the occurrence of pneumonia. The full distribution of the different outcomes is shown in Table 6.

**Table 6:** Outcomes as described in the included studies

Sum	Outcome
25	Nutritional Status
7	Sufficient oral intake
6	Pneumonia
6	Length of Stay
4	Risk of Dysphagia/Swallowing Function
4	Mortality
4	Oral Medication
3	ADL's
2	Place of Discharge
2	Depression/Anxiety
2	Cognitive and Functional Status
2	Rehospitalization
2	Nutritional Risk
1	NIHSS
1	Barthel Index
1	Grip Strength
1	Rehabilitation
1	Weight loss
1	Mobility
1	Frailty
1	Fall

*Legend*: *ADL’s* Activities of Daily Life, *NIHSS* National Institute of Stroke Scale

Only two of the studies explicitly indicated the presence of a specialized nutrition team. Shimazu et al. report the team being led by a dietitian and consisting of doctors, nurses, pharmacists, dentists and SLTs [46]. Waza et al. describe the following professional groups as members of the nutrition team: SLT, registered dietitians, dental assistants, medicine, physiotherapists, occupational therapists, and nursing staff [69]. Furthermore, only Waza et al. report that nutritional counseling takes place systematically upon discharge.

## Discussion

Early detection of malnutrition as well as dysphagia can prevent a cascade of problems resulting from these conditions [15, 30]. Being closely associated conditions, it might be beneficial to always screen for both, since having severe dysphagia will negatively impact nutritional status and vice versa. Guidelines for Geriatric Assessment state that both conditions should be assessed [34, 35, 74]. However, due to time and personnel limitations, this does not always seem to be possible. Looking at the Comprehensive Geriatric Assessment (CGA), for example, feeding and nutrition are not considered part of the core components but mentioned as additional components that may be included [35], hinting at an optional extended version. The British Geriatrics Society (BGS) specifically states in their CGA toolkit for primary care practitioners, that completing the CGA fully “may take up to two hours” but does not provide any specific screening tools for dysphagia aside from asking about swallowing problems, choking or “food getting stuck” [34]. To screen for malnutrition, the BGS recommends monitoring BMI or using the Malnutrition Universal Screening Tool (MUST) [74] or the MNA. In contrast, the living guideline by the German, Austrian and Swiss geriatric societies [75] do recommend three specific screening tools for dysphagia: the SSA (Standardized Swallowing Assessment) [76], the Daniels Water Test [77] and the DSTG (Deutsches Screening-Tool Geriatrie) [78]. Each of them uses solely the application of water in different amounts and with different scales. None of them were reported to be used in the studies included in this review, which in the case of the DSTG is not surprising as it is only available in German. To screen for malnutrition, the guideline recommends the MST (Malnutrition Screening Tool) [79], the MNA and the NRS, with the latter two having been used in several of the studies included in this review.

A screening protocol that combines both disorders and is feasible for different geriatric settings could improve prevention of complications resulting from either condition. As the MNA has excellent sensitivity and specificity (96% and 98% respectively) it might be, especially in its short form, a feasible tool to combine with a swallowing screen. The EAT-10, which has been reported as the sole dysphagia screening tool in eight studies in this review, has the advantage of being self-administered, thus being very feasible for routine use with sensitivity of 85% and specificity of 82% when using a cut-off value of three as recommended by the authors [66, 80]. The downside of a self-administered tool is that language barriers, reduced reading skills, hearing loss and cognitive impairment might make it harder to conduct than simple water swallowing screens [81].

An easy to conduct and fast screening tool should bridge the gap between time and funding constraints and optimal care resulting from early detection of disorders that impact the overall well-being of older people and especially multimorbid geriatric patients. It should contain questions about swallowing ability as well as a quick orientation about nutritional status. The MNA-SF combined with the EAT-10 might offer such an overview. An alternative to the EAT-10 could be the 4QT (4-point questionnaire test), an even shorter questionnaire focusing on changes in swallowing and eating ability [82], which was designed specifically for older and frail people. A limiting factor is that it has not yet been validated against an instrumental examination. In older people who are not able to complete these self-reported questionnaires, the Minimal Eating Observation Form – version II (MEOF-II) could be used alternatively. It focuses on the whole process of eating rather than specifically on the risk of penetration or aspiration, but can be conducted without verbal language skills [83]. As an addition or alternative to the MNA-SF, the SNAQ (Simplified Nutritional Appetite Questionnaire) could be considered. The SNAQ consists of four items and shows a sensitivity of 82% as well a specifity of 85% for a 6-months weight loss of 5% [84].

This scoping review aimed to find out whether any screening protocol combining dysphagia and malnutrition is already in use internationally. Further investigations focus on which professions are involved and which screening tools are used in screening for malnutrition and dysphagia, and the possible outcomes as well as measures derived in the case of reported screening protocols.

The included studies vary widely in describing the use of the respective screenings and the measures taken from the screening results. Due to the large number of cross-sectional studies that assessed the prevalence and association between malnutrition and dysphagia rather than the impact of implementing a systematic screening, only the first part of the research question can be answered. The search results did not yield sufficient studies to answer the questions of how implementing such protocols impacted the outcomes described in the PICO question – namely pneumonia, falls, rehospitalization, length of stay, place of discharge, nutritional status, wound and bone healing, mobility, sufficient oral intake, oral medication, or bolus death.

The included studies are spread globally with the majority from Japan and Spain. It could be speculated that Japan has a high life expectancy for years [85, 86], possibly leading to a stronger research focus on the elderly. However, the wide range of countries illustrates that the topic itself seems to be of worldwide interest. All included studies report the use of dysphagia screening as well as a malnutrition screening, but none have a single combined protocol. The settings of the studies vary, with most conducted in acute care hospitals. In terms of dysphagia screening, various instruments were used, such as the EAT-10, V-VST, FOIS, FILS, MASA, STAND, GUSS and MDADI, as well as water swallowing tests without further description (Table 4). The MNA, either in long or short form, was the most frequently used screening tool for malnutrition, with other instruments such as GNRI, NRS and SGA also being reported. Moreover, this review surveyed the performing professional groups, naming speech therapists, nursing staff and, in some cases, a multi-professional team, but most papers did not report on the professional group carrying out the screenings.

Only a few studies report repeated screening as part of routine care, either after an intervention, after a certain time or before discharge. Measures derived from abnormal screening results, such as referrals to speech therapy, decisions about oral feeding options, and dietary counseling, were documented in 10 of the studies, as shown in Table 5. Overall, the measures reported in the studies vary broadly from simple texture modified diet prescriptions that have been internationally standardized by the International Dysphagia Diet Standardization Initiative (IDDSI) [87] to more complex interventions by nutritional teams. The mentioned nutritional teams comprised several professions and interventions such as speech therapy, counselling by a dietitian, positioning, promotion of oral health, nutritional therapy, respiratory therapy, and medication evaluation, as described in Waza et al. [69] and Shimazu et al. [46].

The most reported outcomes were nutritional status, occurrence of pneumonia, and sufficient oral intake. The distribution of the different outcomes varies, with nutritional status being by far the most frequent. While numerous outcomes were assessed in some studies, others were limited to one or two parameters. Only Waza et al. [69] and Shimazu et al. [46] described an interprofessional nutrition team of different professional groups. Systematic nutritional counseling at discharge was only described by one study [69].

The heterogeneity of the studies in terms of settings, screening instruments and performing professional groups makes direct comparisons between studies difficult. The issue of required training and possible user groups for specific screening tools might also be of interest, as well as the different settings in which the discussed studies were conducted. Examining the extent to which these differences affect the reported results might be intriguing. In addition, the implications of the screening results and the measures derived are not uniformly documented, making it difficult to draw clear conclusions regarding to the questions of this review.

It should be noted that by conducting a scoping review, a lot of different study designs were included in the selection process. Consequently, the research question can only be answered to a limited extent with the available results. The focus of the included studies estimating the prevalence and associations between malnutrition and dysphagia shows their interdependence. However, it is not possible to make assumptions about the impact of a systematically used combined screening for malnutrition and dysphagia on the originally described outcomes of this review. The mention of a nutrition team as well as systematic nutritional counseling at discharge is reported in only two studies. This does not necessarily mean that such procedures are not present, rather that they have not been systematically reported and evaluated. The prevalence and close association of the two investigated conditions strongly suggest a potential improvement of care through early detection of malnutrition and dysphagia.

Further research could focus on conducting implementation studies to determine the impact of a combined screening protocol on the care pathway of geriatric patients. Subsequently, it might be of interest to assess how and with which impact on health outcomes interdisciplinary nutrition teams can be effectively integrated into the care of older people. In future investigations, the validity of a joint screening tool, for example a combination of SNAQ and 4QT should be evaluated. If a combined screening tool proves to be feasible while maintaining high sensitivity and specificity, its integration into routine care should be evaluated considering the outcomes defined in this scoping review.

## Conclusion

Dysphagia and malnutrition in the elderly have complex relationships that can affect not only the state of health, but also quality of life. A lot of different screening protocols are in use globally with different professions being involved in applying those tools. There was no reported combined screening tool, however both conditions are screened regularly in different geriatric inpatient setting globally. Systematic early detection and clearly defined care pathways potentially have a positive effect on nutritional status, sufficient oral intake, the ability to swallow safely and pneumonia rates. These outcomes were reported in the analyzed studies, however it was not possible to establish whether the implementation of screening protocols did influence those factors. Unfortunately, there is lack of research on the impact of systematically implemented screening protocols to ensure sound, evidence-based guidance on these important issues.

## Data Availability

The datasets used and/or analyzed during the current study are available from the corresponding author on reasonable request.

## Abbreviations

WHO: World Health Organization
SLT: Speech Language Therapist
RD: Registered Dietitian
IDDSI: International Dysphagia Diet Standardization Initiative
NPO: Nil per OS
TMD: Texture Modified Diet
EAT-10: Eating Assessment Tool
FOIS: Functional Oral Intake Scale
FILS: Food Intake Level Scale
GUSS: Gugging Swallowing Screen
MASA: Mann Assessment of Swallowing Ability
MDADI: M.D. Anderson Dysphagia Inventory
PARD: Protocol of Risk Assessment for Dysphagia
STAND: Screening Tool for Neurological Dysphagia
SWAL-QOL: Swallowing Related Quality of Life
WST: Water Swallowing Test
V-VST: Volume-Viscosity Swallowing Test
MNA: Mini Nutritional Assessment
MNA-SF: Mini Nutritional Assessment – Short Form
GNRI: Geriatric Nutritional Risk Index
SGA: Subjective Global Assessment
ADL’s: Activities of Daily Life
NIHSS: National Institute of Stroke Scale
CGA: Comprehensice Geriatric Assessment
4QT: 4-Point Questionnaire Test
SNAQ: Simplified Nutritional Appetite Questionnaire
